# Hindcasting Historical Breeding Conditions for an Endangered Salamander in Ephemeral Wetlands of the Southeastern USA: Implications of Climate Change

**DOI:** 10.1371/journal.pone.0150169

**Published:** 2016-02-24

**Authors:** Houston C. Chandler, Andrew L. Rypel, Yan Jiao, Carola A. Haas, Thomas A. Gorman

**Affiliations:** Department of Fish and Wildlife Conservation, Virginia Tech, Blacksburg, Virginia, United States of America; Clemson University, UNITED STATES

## Abstract

The hydroperiod of ephemeral wetlands is often the most important characteristic determining amphibian breeding success, especially for species with long development times. In mesic and wet pine flatwoods of the southeastern United States, ephemeral wetlands were a common landscape feature. Reticulated flatwoods salamanders (*Ambystoma bishopi*), a federally endangered species, depend exclusively on ephemeral wetlands and require at least 11 weeks to successfully metamorphose into terrestrial adults. We empirically modeled hydroperiod of 17 *A*. *bishopi* breeding wetlands by combining downscaled historical climate-model data with a recent 9-year record (2006–2014) of observed water levels. Empirical models were subsequently used to reconstruct wetland hydrologic conditions from 1896–2014 using the downscaled historical climate datasets. Reconstructed hydroperiods for the 17 wetlands were highly variable through time but were frequently unfavorable for *A*. *bishopi* reproduction (e.g., only 61% of years, using a conservative estimate of development time [12 weeks], were conducive to larval development and metamorphosis). Using change-point analysis, we identified significant shifts in average hydroperiod over the last century in all 17 wetlands. Mean hydroperiods were shorter in recent years than at any other point since 1896, and thus less suitable for *A*. *bishopi* reproduction. We suggest that climate change will continue to impact the reproductive success of flatwoods salamanders and other ephemeral wetland breeders by reducing the number of years these wetlands have suitable hydroperiods. Consequently, we emphasize the importance of conservation and management for mitigating other forms of habitat degradation, especially maintenance of high quality breeding sites where reproduction can occur during appropriate environmental conditions.

## Introduction

Small ephemeral wetlands are ecologically important systems that characterize a variety of landscapes worldwide [1–3]. These wetlands serve as breeding sites for amphibians and aquatic invertebrates and are thus important for linking aquatic and terrestrial environments [4, 5]. In addition to providing breeding habitat, ephemeral wetlands often support unique taxa. Species inhabiting ephemeral wetlands frequently experience tradeoffs between reduced predation pressure and increased risk of mortality from desiccation [6]. Regular drying events extirpate predatory fish populations and reduce the rate at which slow-developing predatory invertebrates become established in ephemeral wetlands, which indirectly lowers predation pressure on other aquatic organisms [7, 8]. However, drying events also increase mortality risk for aquatic species because of desiccation [9]. Larval amphibians require a minimum time period in aquatic environments to successfully metamorphose into terrestrial adults [10], and aquatic invertebrates require a long enough time to reproduce and/or reach a desiccation-resistant stage [11]. Therefore, hydroperiod (i.e., length of inundation between drying events) plays a key role in driving species composition and biological success in most ephemeral wetlands [12].

Hydroperiods in isolated ephemeral wetlands are strongly influenced by precipitation and evapotranspiration [13–15]. For example, in the southeastern United States coastal plain physiographic province, a majority of annual precipitation feeding into ephemeral wetlands can be lost through evapotranspiration [16, 17]. Evapotranspiration rates are often directly related to air temperatures, with higher temperatures leading to higher evapotranspiration rates [18]. Ephemeral wetlands can also serve as sources or sinks for groundwater flow depending on geologic conditions of the surrounding landscape, but this usually has a smaller influence on wetland hydroperiod [19]. The strong influence of short-term weather events on ephemeral wetland hydrology makes these systems susceptible to changes in temperature and precipitation patterns [20].

Pine flatwoods are found along the coastal plain of the southeastern United States in low-lying areas with poorly drained soils [21]. Embedded within this ecosystem are shallow ephemeral wetlands that typically fill with water during fall or winter rains when evapotranspiration rates are low and normally experience a dry period during the spring or summer when evapotranspiration rates are high [22–24]. Climate change models predict that the southeastern United States will likely face longer periods of drought, a more unpredictable rainfall pattern, and higher evapotranspiration rates [25–28]. Climatic changes will undoubtedly alter hydrologic processes in these ephemeral ecosystems [20] and thus represent a significant challenge to species endemic to these environments.

Ephemeral wetlands in pine flatwoods provide breeding habitat for a diverse amphibian assemblage [29, 30]. This includes the federally endangered reticulated flatwoods salamander (*Ambystoma bishopi*) [31]. Adult flatwoods salamanders migrate from surrounding uplands to breeding wetlands on rainy nights from October–December [32, 33]. Females lay eggs in dry wetland basins, and the eggs hatch after they are inundated by winter rains [32, 34, 35]. Larval salamanders then require between 11–18 weeks to fully develop and metamorphose into adults [36]. This relatively long larval period makes flatwoods salamander populations susceptible to complete reproductive failure not only during severe droughts, but also during years when wetlands have a shortened or variable hydroperiod.

Well-documented worldwide amphibian declines have been attributed to many factors including climate change [37, 38]. In ambystomatid salamanders, population declines and reproductive failures can be caused by shortened hydroperiods at breeding wetlands during drought years [39–42]. For example, recent population declines in frosted flatwoods salamanders (*Ambystoma cingulatum*) were linked to complete reproductive failure during consecutive drought years [43, 44]. Furthermore, declines in the adult breeding population can continue to negatively impact reproductive success even during years with favorable breeding conditions [45], and in some ambystomatid salamander populations large percentages of individuals appear to skip migrations to breeding wetlands even during years with adequate rainfall [46].

The goals of this study were to: 1) characterize the hydroperiod of *A*. *bishopi* breeding wetlands during recent years (2005–2014); 2) generate empirical models capable of hindcasting historical hydroperiods of flatwoods salamander breeding wetlands; and 3) identify temporal changes in wetland hydroperiods over the last 119 years. We predicted that historical weather conditions, and therefore historical hydroperiods, were once more favorable (i.e., longer) for successful flatwoods salamander reproduction and that the frequency and severity of drought periods has increased in recent years.

## Materials and Methods

### Ethics Statement

Eglin Air Force Base and U.S. Fish and Wildlife Service granted permission to access field sites. This study was a habitat study, and we did not collect any data on vertebrate animals during the study (only collected data related to wetland characteristics). Therefore, we did not require collecting permits or IACUC approval but did need permission to access study sites.

### Study Sites

Ephemeral wetlands were identified as *A*. *bishopi* breeding sites during surveys conducted in the mid-1990s or early 2000s on Eglin AFB in Okaloosa and Santa Rosa counties, Florida, U.S.A. [33, 47, 48]. We have omitted the exact location of study wetlands because this would reveal the location of sensitive endangered species habitat, but this information is available from the U.S. Fish and Wildlife Service. Eglin AFB is a large U.S.A. military installation with over 146,000 ha of actively managed longleaf pine forests. Beginning in the early 2000s, we have continuously monitored 17 *A*. *bishopi* breeding wetlands [48, 49]. The most recent surveys (2003–2014) have documented *A*. *bishopi* larvae in only 10 of the original 17 wetlands despite increased survey effort, 11 years of survey data, and habitat protection along with some active management on Eglin AFB [49, 50]. Study wetlands typically contained a longleaf pine (*Pinus palustris*) and slash pine (*Pinus elliottii*) overstory with an understory of herbaceous plants, but some wetlands had developed a woody midstory, as a result of historic fire suppression [22, 50]. These wetlands are similar to other southeastern coastal plain wetlands used by breeding flatwoods salamanders, although much of the upland habitat in which they are embedded may be in better condition because of Eglin AFB’s active prescribed fire program. However, even on this actively-managed site, there are a range of conditions related to human modification including fire-suppressed uplands and wetlands, plow lines and ditching from previous fire suppression, runoff and changes to infiltration from nearby roads and development, and encroachment of invasive plants [49, 51, 52].

### Data Collection

Based on accumulated experience from sampling the 17 wetlands for *A*. *bishopi* larvae, a metal stake was anchored at the approximate center of each wetland. We measured water depth at this point in each wetland from November 2005–May 2014 (S1 Table). We generally recorded measurements twice a month, but water levels were occasionally measured monthly. We used the recorded depth measurements to characterize wetland hydroperiods from 2006–2014 (hereafter the year will refer to the January–May portion of each breeding season). We identified all instances of wetland filling and drying and identified the longest yearly hydroperiod that occurred at least partially during the *A*. *bishopi* breeding season (November–May). Dates outside of this monthly range were included if there was a continuous hydroperiod that started before or ended after the breeding season. We used these observed yearly hydroperiods to calculate an average hydroperiod for each wetland and mean and median filling and drying dates. We also calculated a maximum observed depth (based on our measurements) and maximum wetted area (using GPS) for each wetland (S2 Table).

We used modeled, historic downscaled climate data for our study sites from the PRISM climate database (Oregon State University, http://prism.oregonstate.edu, created 3 February 2015). Briefly, PRISM uses climatologically-aided interpolation to predict long-term (1896–2014) downscaled climate normals for a geographic point of interest. Ultimately, modeled climate conditions for a geographic point of interest are based on interpolations of climate data from whatever weather stations and data sources are available for the period of interest. We used PRISM climate data for a single geographic coordinate (30.423, −86.771), representing the center of our study area. All 17 wetlands included in our analysis were within 11 km of this location. The PRISM dataset contained monthly estimates of precipitation totals, monthly maximum temperature, and monthly minimum temperature. We also included all three climate variables lagged by one month because the previous month’s climate conditions might have a large influence on water levels. Finally, we used the Palmer Hydrological Drought Index (PHDI; National Oceanic and Atmospheric Administration, http://www.ncdc.noaa.gov/) for the northwest portion of Florida (1896–2014). This index measures the long-term hydrological impacts of drought (e.g., groundwater levels). Only data from November–May were included in the final analyses because by November most adult flatwoods salamanders would have migrated to breeding sites, and recent data suggest that larval salamanders may be present during May in years where wetlands fill or retain water later than normal (Haas and Gorman, unpublished data).

### Statistical Analyses

We converted depth measurements into a binary code where months in which wetlands held water were coded as 1, and months where wetlands had no water at the center stake were coded as 0 (months with two measurements were coded as 0 if there was no water at the center stake during either measurement because a single drying event would likely cause mortality for larval salamanders). We used a principal component analysis (PCA) to reduce the four temperature variables to a few principal components (PCs). Only principal components with an eigenvalue ≥1 were selected for inclusion in our models. Prior to building models we scaled the precipitation, wetland depth, and wetland area data by subtracting the mean and dividing by the standard deviation.

We fit generalized linear mixed models (GLMMs) to relate climatological and wetland-specific characteristics to the presence/absence of water in wetland basins on a monthly basis (binomial error distribution and logit link function). Our predictor variable set (fixed effects) included precipitation, lagged precipitation, temperature (summarized in PCs), PHDI, wetland area, and wetland depth. Month was also included in the model to account for additional temporal variation. We developed three broad *a priori* hypotheses that the presence of water would be related to 1) short-term climate data (temperature and precipitation), 2) the PHDI, or 3) a combination of short-term climate data and the PHDI. For each hypothesis, we fit eight models that also included wetland-specific variables (area and depth) and temporal variables (month). Our candidate model-set contained 24 candidate models (Table 1). We included wetland ID as a random effect in each model to account for the repeated measures in each wetland and to make site-specific predictions (all models were fit with only random intercepts).

**Table 1:** Generalized linear mixed models.

Model	AIC	ΔAIC	*w*_*i*_	K
Lprecip. + Precip. + Temp. + PHDI + Area + Month	840.6	0.0	0.61	14
Lprecip. + Precip. + Temp. + PHDI + Area + Depth + Month	841.9	1.3	0.31	15
Lprecip. + Precip. + Temp. + PHDI + Month	845.3	4.7	0.06	13
Lprecip. + Precip. + Temp. + PHDI + Depth + Month	847.2	6.6	0.02	14
Lprecip. + Precip. + Temp. + PHDI + Area	858.2	17.6	0.00	8
Lprecip. + Precip. + Temp. + PHDI + Area + Depth	859.6	19.0	0.00	9
Lprecip. + Precip. + Temp. + PHDI	863.0	22.4	0.00	7
Lprecip. + Precip. + Temp. + PHDI + Depth	864.9	24.3	0.00	8
Lprecip. + Precip. + Temp. + Area + Month	865.8	25.3	0.00	13
Lprecip. + Precip. + Temp. + Area + Depth + Month	867.2	26.6	0.00	14
Lprecip. + Precip. + Temp. + Month	870.6	30.0	0.00	12
Lprecip. + Precip. + Temp. + Depth + Month	872.5	31.9	0.00	13
Lprecip. + Precip. + Temp. + Area	918.6	78.0	0.00	7
Lprecip. + Precip. + Temp. + Area + Depth	919.9	79.3	0.00	8
Lprecip. + Precip. + Temp.	923.4	82.8	0.00	6
Lprecip. + Precip. + Temp. + Depth	925.3	84.73	0.00	7
PHDI + Area + Month	1113.3	272.7	0.00	10
PHDI + Area + Depth + Month	1114.8	274.2	0.00	11
PHDI + Month	1118.5	277.9	0.00	9
PHDI + Depth + Month	1120.4	279.9	0.00	10
PHDI + Area	1129.8	289.2	0.00	4
PHDI + Depth + Area	1131.3	290.7	0.00	5
PHDI	1135.0	294.4	0.00	3
PHDI + Depth	1136.9	296.3	0.00	4

Model set and results relating the monthly presence/absence of water in pine flatwoods wetlands used by *Ambystoma bishopi* for breeding on Eglin Air Force Base, Florida to climatologic, temporal, and site-specific factors (AIC = Akaike’s Information Criterion, ΔAIC = change in AIC, *w*_i_ = relative amount of support for each model, and K = the number of parameters). Wetland ID was included as a random intercept in each model, and temperature variables were summarized in two principal components. PHDI represents the Palmer’s Hydrologic Drought Index, and Lprecip represents the previous month’s precipitation.

We used an information theoretic approach and Akaike's Information Criterion (AIC) to examine the strength of *a priori* hypotheses. We considered the model with the lowest AIC to have the best balance of statistical parsimony and goodness of fit for the data [53]. We used the best-approximating model to evaluate whether or not each wetland was wet or dry during each month of every *A*. *bishopi* breeding season from 1896–2014. We validated model output with a validation dataset that contained monthly water-level measurements from previous years. These other water-level measurements were collected independently from a subset of sites during *A*. *bishopi* surveys conducted from 1993–1994 [47] and 2003–2005 [48].

We used hindcasted wet/dry predictions from the best-approximating model to calculate hydroperiods for each wetland in every breeding season from 1896–2014. Hydroperiods were calculated by adding the longest consecutive number of wet months during each year. We then used estimated hydroperiods to determine the number of years that would be unsuitable for *A*. *bishopi* reproduction. An unsuitable breeding year was defined as a year where the hydroperiod was too short for larval salamanders to successfully metamorphose based on three different lengths of time: 3 months, 4 months, and 5 months [36]. Lastly, we performed a change-point analysis on the estimated hydroperiods for each wetland. Change-point analyses identify points in time-ordered data where the statistical properties of those data have changed [54]. We used the Pruned Exact Linear Time (PELT) method [55] to identify change points. We only reported time periods between change points that were longer than 10 years, and all change points were calculated with a theoretical type I error rate of 0.05. All analyses were performed in R [56]. We fit GLMMs using the lme4 package [57], and we calculated AIC values and model weights using the package AICcmodavg [58]. Change points were identified using the R package changepoint [59].

## Results

**Fig 1:**
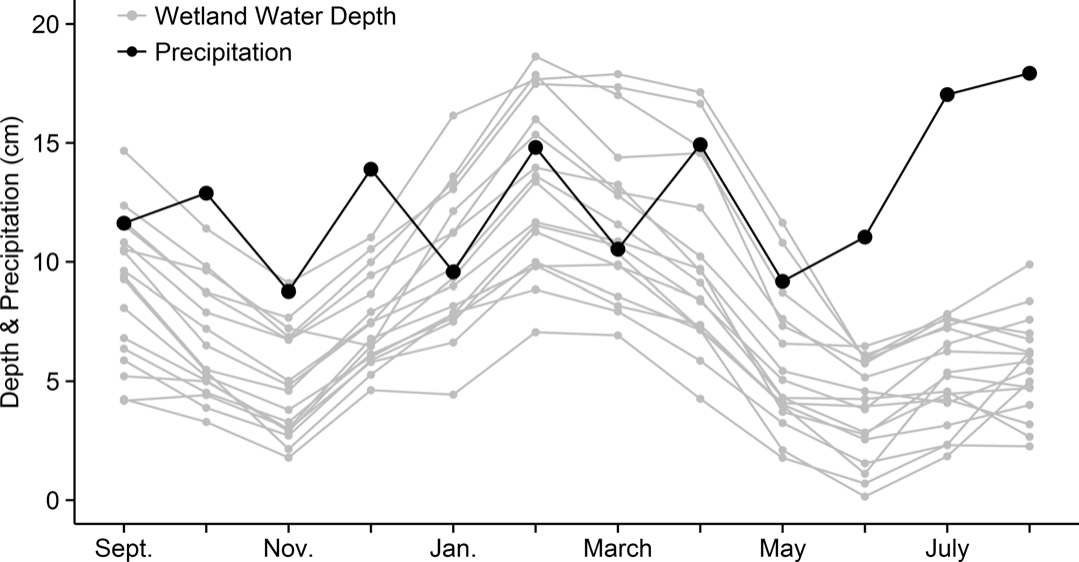
Average monthly water depth and precipitation in pine flatwoods wetlands on Eglin AFB in northwest Florida. Average monthly water depth of 17 pine flatwoods wetlands (used for breeding by *Ambystoma bishopi*) from 2005–2014 versus the average monthly precipitation over the same time period.

**Fig 2:**
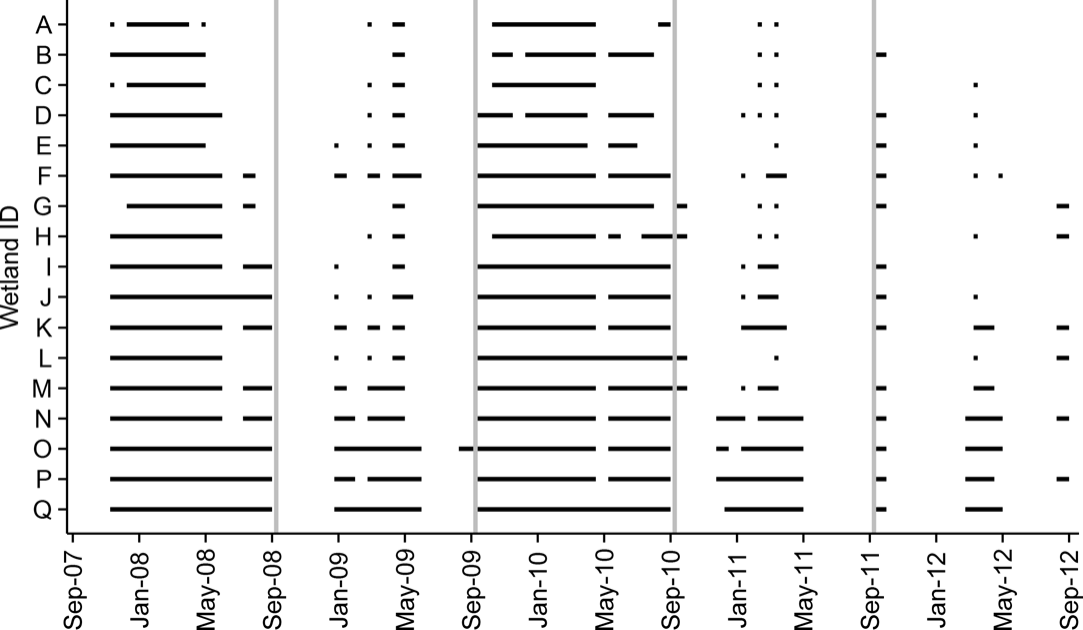
Hydroperiods in pine flatwoods wetlands on Eglin AFB in northwest Florida. Measured hydroperiods from 2007–2012 for 17 pine flatwoods wetlands used by *Ambystoma bishopi* for breeding. Depth measurements used to calculate hydroperiods were recorded at an approximate center point in each wetland. Years are arranged from September–August, and vertical gray lines separate years.

Over a 9-year period from 2006–2014, wetlands were usually deeper during the breeding season despite an increase in precipitation during summer months (Fig 1). Wetland fill dates varied from year to year. However, average fill dates occurred during December or January, while average dry down dates generally occurred during April or May (Table 2). Observed hydroperiods also varied among wetlands and across years (Fig 2). Multiple *A*. *bishopi* breeding seasons were characterized by severe drought with the worst occurring from 2006–2007 (all wetlands dry for 18 months), but there were also years where some wetlands were full for the entire *A*. *bishopi* breeding season (Fig 2).

**Table 2:** Filling and drying dates for pine flatwoods wetlands on Eglin AFB in northwest Florida.

Wetland	Mean Hydroperiod	Date of Filling		Date of Drying	
ID	(Months)	Mean	Median	Mean	Median
A	2.3	Jan. 16	Feb. 8	Apr. 21	Apr. 21
B	2.4	Jan. 13	Feb. 8	Apr. 16	Apr. 8
C	2.6	Jan. 17	Jan. 16	Apr. 19	Apr. 3
D	2.7	Jan. 18	Feb. 18	Apr. 21	Apr. 3
E	2.8	Jan. 19	Jan. 29	Apr. 25	Apr. 15
F	3.7	Jan. 21	Feb. 8	Apr. 25	Apr. 21
G	3.9	Dec. 22	Jan. 5	May 28	May 27
H	3.9	Jan. 8	Feb. 18	May 26	May 3
I	4.0	Dec. 15	Dec. 31	Apr. 29	Apr. 16
J	4.1	Jan. 3	Feb. 5	May 21	May 13
K	4.2	Dec. 22	Jan. 19	Jun. 5	May 27
L	4.2	Jan. 1	Jan. 29	May 24	Apr. 15
M	4.2	Dec. 26	Jan. 29	May 20	May 11
N	4.5	Dec. 25	Jan. 29	May 26	May 19
O	4.9	Dec. 16	Dec. 25	May 30	May 18
P	5.1	Dec. 10	Dec. 31	Jun. 1	May 22
Q	5.9	Dec. 9	Dec. 16	Jun. 28	Jun. 12

Mean and median dates of filling and drying for 17 pine flatwoods wetlands used by *Ambystoma bishopi* for breeding. Water levels were measured at an approximate center point from December 2005–May 2014. The range of fill dates was September 1–April 8, and the range of dry dates was January 21–October 21 of the following fall.

Temperature data were summarized into two principal components that explained 97.2% of the variation in the data (Table 3), and these two PCs were included in the GLMMs (PC1 = 1.7, 73.2%; PC2 = 1.0, 24.0%). The first PC was negatively correlated with all four temperature variables, and PC2 was positively correlated with the monthly temperature but negatively correlated with the previous month’s temperature (Table 3). All other climatological variables were not strongly correlated (all |r| ≤ 0.4). Our modeling results indicated that a combination of short-term climate data and PHDI were better predictors of wetland conditions (wet or dry) than either short-term climate data or PHDI alone (Table 1). In addition to climate data and PHDI, the best-approximating model contained variables for wetland area and month (Table 1). Precipitation, previous month’s precipitation, PC1, PHDI, and area all had a positive influence on the presence of water in a wetland (Table 4). Using independent water-level data as a validation dataset, the best-approximating model correctly classified 98 out of 133 (73.7%) months as being wet or dry.

**Table 3:** Principal component analysis of temperature variables.

	PC1	PC2
Max Temp (°C)	−0.52	0.45
Min Temp (°C)	−0.49	0.54
Lagged Max Temp (°C)	−0.48	−0.55
Lagged Min Temp (°C)	−0.51	−0.46

Factor loadings for two principal components from a Principal Component Analysis of four temperature variables. Eigenvalues were 1.7 and 1.0 for principal components 1 and 2 respectively. Other principal components were not included because their eigenvalues were less than 1.0.

**Table 4:** Parameter estimates for the best-approximating model.

Variable	Coefficient	SE	95% CI
Precipitation	1.28	0.15	0.99–1.57
Lagged Precipitation	1.62	0.15	1.32–1.93
PC1	0.63	0.16	0.32–0.94
PC2	−0.13	0.19	−0.50–0.24
PHDI	0.26	0.05	0.16–0.36
Area	0.48	0.17	0.15–0.81
November	0.11	0.63	−1.13–1.34
December	−0.13	0.38	−0.86–0.61
January	−0.33	0.43	−1.18–0.51
February	−0.45	0.33	−1.09–0.19
March	0.26	0.44	−0.61–1.12
April	2.10	0.61	0.90–3.29
May	2.77	0.86	1.08–4.46

Parameter estimates, standard errors (SE), and 95% confidence intervals for the fixed effects in a generalized linear mixed model predicting whether or not pine flatwoods wetlands used for breeding by *Ambystoma bishopi* hold water in a given month. The model included data from 2006–2014 from 17 pine flatwoods wetlands on Eglin Air Force Base, Florida.

**Fig 3:**
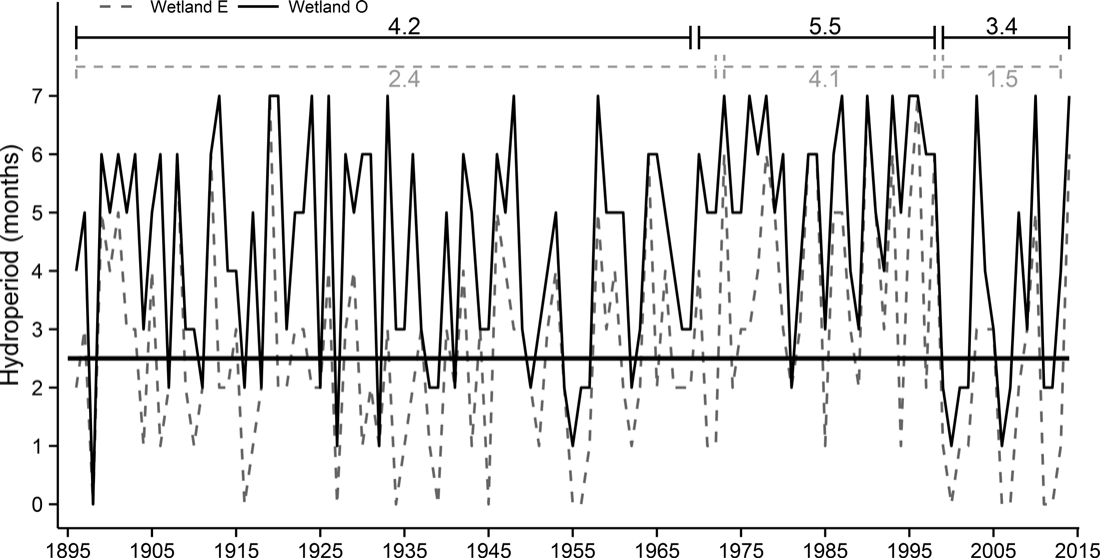
Historical hydroperiods modeled in pine flatwoods wetlands. Estimated hydroperiods (November–May) from 1896–2014 for two pine flatwoods wetlands (wetland E and O), on Eglin AFB, Florida, used for breeding by *Ambystoma bishopi* based on generalized linear mixed model predictions of wetland conditions. Change points in average hydroperiod occurred around 1970 and 1999. Change points are indicated by lines above the graph, and the average hydroperiod between each change point is indicated along these lines. The horizontal black line represents the hydroperiod below which *A*. *bishopi* cannot successfully reproduce.

**Fig 4:**
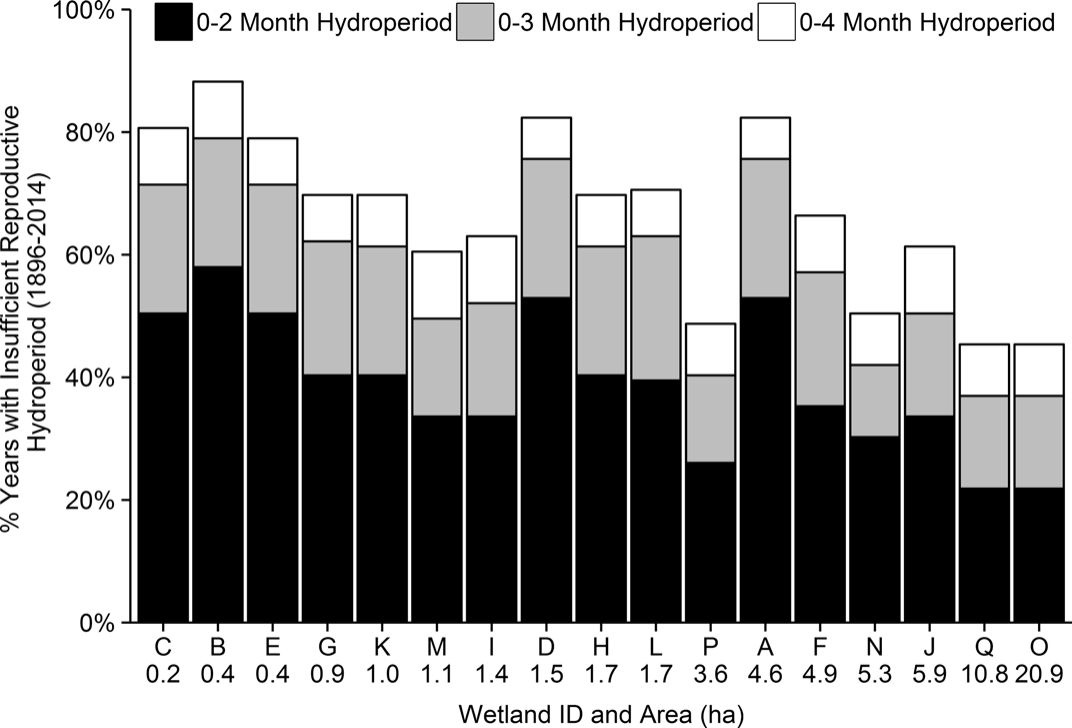
Suitability of hydroperiods for flatwoods salamander reproduction. Percentage of years from 1896–2014 in which the hydroperiod of 17 pine flatwoods wetlands was likely insufficient for *Ambystoma bishopi* reproduction based on larval development times of between 3 and 5 months. Each bar represents a different breeding wetland arranged in order of increasing wetted area.

Estimated hydroperiods over the past nine breeding seasons were not significantly different from measured hydroperiods over the same time period (2.5 [SE = 0.20] months compared to 2.8 [SE = 0.22] months; t_304_ = 1.22; *P* = 0.31). The average estimated wetland hydroperiod from 1896–2014 ranged from 2.3–4.4 months, which reflected the variation among wetlands. In addition to the variation among wetlands, there was also substantial variation in estimated hydroperiods through time (illustrated for two wetlands in Fig 3). Between 39–67% of years from 1896–2014 were likely unsuitable for flatwoods salamander reproduction depending on larval development times (Fig 4). In addition to the overall variability in hydroperiod, we detected a change in average hydroperiod in all 17 wetlands (Table 5). All wetlands shifted to a longer average hydroperiod around either 1950 or 1970. However, this period of longer hydroperiods was followed by shortening of hydroperiods in all wetlands after 1999 (Fig 3; Table 5). Average hydroperiods after this second shift were, on average, 0.8 months shorter than at any time during the last 119 years.

**Table 5:** Results of change-point analysis on wetland hydroperiods.

	1^st^ Change Point		2^nd^ Change Point		3^rd^ Change Point	
Wetland ID	Year	Mean	Year	Mean	Year	Mean
A	1896–1972	2.32	1972–1998	3.88	1999–2013	1.47
B	1896–1972	2.09	1973–1998	3.62	1999–2013	1.20
C	1896–1972	2.44	1973–1998	4.12	1999–2013	1.53
D	1896–1972	2.32	1973–1998	3.92	1999–2013	1.47
E	1896–1972	2.44	1973–1998	4.12	1999–2013	1.53
F	1896–1972	3.13	1973–1998	4.65	1999–2014	2.56
G	1896–1972	2.92	1973–1998	4.54	1999–2014	2.13
H	1896–1972	2.84	1973–1998	4.42	1999–2014	2.13
I	1896–1953	3.38	1958–1998	4.41	1999–2014	2.75
J	1896–1953	3.43	1958–1998	4.44	1999–2014	2.75
K	1896–1972	2.96	1973–1998	4.58	1999–2014	2.13
L	1896–1972	3.00	1973–1998	4.58	1999–2014	2.13
M	1896–1953	3.48	1958–1998	4.46	1999–2014	2.81
N	1896–1948	4.13	1958–1998	4.88	1999–2014	2.94
O	1896–1969	4.20	1970–1998	5.48	1999–2014	3.38
P	1896–1971	4.07	1972–1998	5.33	1999–2014	3.06
Q	1896–1969	4.20	1970–1998	5.48	1999–2014	3.38

Change points in the mean hydroperiod from 1896–2014 for 17 pine flatwoods wetlands used for breeding by *Ambystoma bishopi* on Eglin Air Force Base, Florida. Reported periods between change points were longer than 10 years (i.e., some short periods were omitted), and all change points had a theoretical type I error rate of 0.05.

## Discussion

This is the first study to examine hydroperiod fluctuations in ephemeral wetlands of the southeastern United States over the past century. Model predictions indicated that all 17 wetlands experienced multiple shifts in average hydroperiod during the past 119 years, and the shortest average hydroperiods occurred over the past 15 years. Changes in hydroperiod were driven by changes in precipitation and drought conditions during the *A*. *bishopi* breeding period (e.g., average PHDI from 1896–1976 = −0.06, from 1977–1999 = 0.64, and from 2000–2014 = −0.99). Shifts in ephemeral wetland hydroperiods are important because hydroperiod is often the most important characteristic driving species composition and breeding success of aquatic organisms, especially for slow-developing amphibian species like flatwoods salamanders [9, 10, 12, 52]. These wetlands provide essential breeding habitat to diverse plant and animal communities that depend on yearly wetting and drying cycles [4, 5].

Shortened hydroperiods coincided with a recent decline in the number of wetlands occupied by *A*. *bishopi*, despite increased survey effort [48, 49]. The average hydroperiod across all wetlands during the initial flatwoods salamander surveys (1993–1994) was 4.4 months compared to just 2.8 months from 2003–2014 (when current surveys were conducted). We also documented one breeding season (2006–2007) where none of the breeding wetlands filled and several other years where hydroperiods in most wetlands were unsuitable for *A*. *bishopi* reproduction. Mean and median fill dates over the past nine years also occurred surprisingly late in the season (median dates in late January to early February), which indicates that in many breeding seasons salamanders may only have a 2–3 month window before evapotranspiration rates increase in April and May (if wetlands fill at all). Furthermore, others have documented complete reproductive failure and decreases in the adult population size of *A*. *cingulatum* populations after consecutive drought years during the early 2000s [43, 44]. Similarly, severe droughts leading to pond drying increased local extinction probabilities for mole salamanders (*Ambystoma talpoideum*) in northern Florida during the same time period [42]. These results support the hypothesis that an increased frequency and severity of drought has contributed to a reduction in wetlands occupied by breeding *A*. *bishopi* on Eglin AFB.

In addition to shorter hydroperiods and a reduction in occupied breeding wetlands in recent years, estimated hydroperiods indicated that between 39–67% of breeding seasons over the past 119 years have likely been insufficient for flatwoods salamander reproduction [36]. Like other ephemeral wetland specialists, *A*. *bishopi* populations can persist through drought years when complete reproductive failure can occur, as has been documented during recent years (Fig 2) [49]. During years with little or no recruitment, any reduction in adult survival rates would have severe negative impacts on the ability of populations to persist [40, 43]. Little is known about adult flatwoods salamanders after they emigrate from breeding wetlands, and information on habitat requirements, foraging success, and survival in the uplands (especially with rising temperatures) deserves further study. However, in captivity, adult flatwoods salamanders lived for no longer than four years [60]. Even though other species of ambystomatid salamanders can live longer [61], some studies suggest that many adult ambystomatids may only breed once [41, 62]. A short adult life span, potential negative effects of increased temperature on growth and survival [63], and generally low reproductive output across ambystomatid salamanders increases the susceptibility of flatwoods salamander populations to increasing drought severity and habitat changes that negatively affect larval, juvenile, or adult survival.

The frequency and severity of drought across the southeastern United Stated is projected to increase over the next century as a result of rising temperatures [26, 64]. In northern Florida, both minimum and maximum temperatures are projected to increase by 1.7° to 2.0°C over the next century, which will increase evapotranspiration rates [24, 28]. This region is also projected to experience an increase in total rainfall over the next century, but this increased rainfall is likely to come more unpredictably during severe weather events [25, 26, 28, 64]. Overall, given the percentage of dry years since 1896 and the shortening of hydroperiods after 1999, it is likely that any benefits of increased precipitation will be offset by less predictable hydroperiods and a higher frequency of years with reproductive failure. The predictability of hydroperiods is likely more important for flatwoods salamanders, which select egg deposition sites before wetlands fill, than for other ambystomatid salamanders that rely on the presence or depth of water to identify breeding sites [35].

Precipitation and evapotranspiration rates are often the most important factors influencing the hydrology of small, isolated ephemeral wetlands [13, 15]. The importance of evapotranspiration rates is reflected in the summer drying events despite an increase in precipitation over the same period. Increasing temperature (PC1) also had a negative effect on a wetland’s ability to hold water in the best-approximating model (PC1 negatively correlated with min and max temperature). The inclusion of the PHDI in the top models indicates that current water table heights are often integrating climatological and environmental processes occurring over periods greater than one or two months. Recent data suggest that some pine flatwoods wetlands lose surface water through groundwater loss, which increases drying rates (Chandler, Gorman, Haas, and McLaughlin unpublished data). Even though we did not specifically account for this in our models, wetland area appears to be a driver in the rate of groundwater loss, and wetland area was included in the top model. Overall, using a combination of climatological data and site-specific factors allowed us to accurately classify when wetlands would hold water and generate long-term hydroperiod predictions that would not have been possible using other data sources.

There are several factors that could have impacted the accuracy of our models. First, water levels were measured at only a single approximate center point in each wetland, which did not necessarily correspond to the deepest point in the wetland. It is possible that water was present in small amounts in other parts of the wetland but not at the water-level stake. Second, the data collected for our models were only collected twice a month at most, and monthly climate data were used to build models. The resolution of the climate data and the measured water levels means that our models cannot make predictions on finer spatiotemporal scales. For example, ephemeral wetlands do not necessarily fill or dry at the beginning or end of the month even though our predictions suggest this dynamic. Third, it is possible that larval salamanders are able to survive through a drying event by retreating into crayfish burrows or other similar holes that provide access to groundwater [65], provided drying events had a short duration. Finally, there are other factors that can influence the hydroperiod of ephemeral wetlands that were not included in our models. For example, vegetation structure can influence evapotranspiration rates, and a history of fire suppression has changed the vegetation structure in many of the study wetlands [50]. There is no record of vegetation structure in the study wetlands during the late 1800s and early 1900s. Extensive fire suppression was uncommon until the 1930s, and fire was still a common tool used to manage forests during the early 1900s [66]. If the wetlands and surrounding uplands contained fewer shrubs and trees than they do now, those wetlands could have had even longer hydroperiods pre-1930 than predicted by our models.

The modeling approach used in this paper could be useful for other ecosystems and taxonomic groups. Ephemeral wetlands or ecologically similar habitats (e.g., ephemeral streams and floodplains) occur in myriad landscapes subjected to climatic variability [2, 20]. There are many organisms, including other amphibians breeding in ephemeral wetlands [5, 41, 45, 52], fish inhabiting floodplain lakes [67], aquatic invertebrates in ephemeral habitats [11], and even wetland plants [68] that face similar ecological challenges to the ones described in this paper. More generally, suitable breeding conditions could be modeled using similar techniques for any species that depends, at least in part, on some optimum set of climatic circumstances [69]. Linking historical climatological variations to periods of time when a species experiences suitable breeding conditions will allow managers to better understand how future climate changes are likely to impact populations.

## Conclusions

The frequency of years with an unsuitable hydroperiod for *A*. *bishopi* reproduction implies that populations are susceptible to habitat changes that reduce recruitment or adult survival [43]. The continued and increasing influences of climate change across the southeastern United States will likely intensify this effect. Longleaf pine forests are one of the most endangered ecosystems in the world, and there are numerous anthropogenic changes that can negatively impact breeding and upland habitat including fire suppression, logging, sedimentation, altered hydrology from ditching or well-drilling, road construction, and urbanization [50, 70, 71]. However, some of these same negative impacts could be reconsidered within a climate change adaptation context so as to mitigate negative effects on sensitive taxa [72]. A long history of fire suppression and the inability of winter fires to burn through wetland basins (prescribed burns are typically set during winter months when basins are inundated) has altered the vegetation structure in many of the remaining pine flatwoods wetlands [22, 51]. Therefore, conservation efforts could focus on natural vegetation management as a tool for mitigating climate change impacts on flatwoods salamanders. By ensuring wetland basins are regularly burned and potentially removing woody vegetation from overgrown wetlands, hydroperiods might be lengthened to favor sensitive taxa like the flatwoods salamander during critical periods [50, 73].

Increasing spatial and temporal variation in ephemeral wetland hydroperiods is also generally considered to be an important conservation management strategy [4, 74]. However, for flatwoods salamanders, preserving ephemeral wetlands with longer hydroperiods is likely more important than supporting a diversity of wetland hydroperiods. Breeding in wetlands with longer hydroperiods (as long as they remain ephemeral) may increase the chances of some successful reproduction even during years with moderate hydroperiods. It is unlikely that even wetlands experiencing the best management practices will have suitable hydroperiods during severe drought years, and many ephemeral wetlands are likely to experience prolonged droughts over the next 45 years [75]. Thus, we recommend that managers focus on maintaining or restoring breeding wetlands that have high quality vegetation structures, unaltered hydrology, and support the longest possible hydroperiod (while still remaining ephemeral) during moderate years. Wetland area had a positive effect on a wetland’s ability to hold water and larger wetlands tended to have longer hydroperiods, although there were exceptions and our sample size was small. Even low recruitment in a few wetlands during moderate years reduces the potential for prolonged droughts to result in local extirpations. Finally, managing pine flatwoods landscapes to support wetlands that are not isolated from each other increases the chances that a portion of the population can persist through severe droughts to recolonize wetlands that experience local extirpations.

## Supporting Information

S1 Table Water depth measurements from 17 pine flatwoods wetlands over a 9-year period. (XLSX)

S2 Table Area and maximum depth (2005–2014) of 17 pine flatwoods wetlands. (XLSX)
